# Chinese herbal medicine promotes growth by improving nutrient utilization and rumen microbiota in suckling lambs

**DOI:** 10.3389/fmicb.2025.1644331

**Published:** 2025-10-07

**Authors:** Yan Wang, Yinglian Wu, Rongyan Qin, Xiangyu Chen, Limeng Liu, Lele Wang, Wenqi Wang, Yanfeng Liu

**Affiliations:** ^1^Feed Research Institute, Xinjiang Uygur Autonomous Region Academy of Animal Science, Ürümqi, China; ^2^Xinjiang Key Laboratory of Herbivorous Livestock Feed Biotechnology, Ürümqi, China

**Keywords:** compound Chinese herbal medicine, lamb, rumen fermentation, rumen microbiota, carbohydrate-active enzymes

## Abstract

This study investigated the effects of compound Chinese herbal medicine (CCHM) on nutrient digestibility, rumen fermentation parameters, and microbial structure in suckling lambs. Sixty Lambs born as twins (from the same ewe), each 8 days old, were randomly assigned to two groups. The control and treatment groups received 0 and 0.2% CCHM in the basal diet, respectively. Digestion experiments were conducted during the trial. Rumen fluid samples were collected from slaughtered lambs in the final week for microbiome analysis. The results indicated that average daily gain and average daily feed intake were significantly improved by CCHM. The apparent digestibility of dry matter and acid detergent fiber also increased significantly. CCHM supplementation elevated Ammonia nitrogen (NH3-N), total volatile fatty acids (TVFAs), acetate, and propionate concentrations in the rumen. The relative abundance of Firmicutes, Actinobacteria, Patescibacteria, *Succiniclasticum, Selenomonas, Olsenella*, and *Shuttleworthia* increased in the treatment group. Linear discriminant analysis Effect Size (LEfSe) revealed ten bacterial groups significantly enriched in the treatment group. These included Patescibacteria (Phylum), Negativicutes and Saccharimonadia (Class), Saccharimonadia and Rhodobacterales (Order), Saccharimonadiahe and Rhodobacteraceae (Family), and *Prevotell-9, Saccharimonadales*, and *Limosilicobacillus* (Genus). Thirteen CAZyme families were detected. Two enzyme families, GH34-5 and CBM4, were enriched in the control group, while eleven families were enriched in the treatment group: GT14, GH89, GH84, GH63, GH5-36, CBM58, PL37, GH85, GH165, GH110, and GH50. Correlation analysis between rumen bacteria, carbohydrate enzymes, and fermentation parameters showed a positive correlation between *Saccharimonadales* and GH63. *Limosilactobacillus* showed a positive correlation with CBM58. Negative correlations were found between *Romboutsia* and both GT14 and PL37. GH84, GH165, GH85, and GH50 were positively correlated with NH3-N concentration. CBM58, GT14, GH89, GH110, GH50, and PL37 showed positive associations with TVFAs. This study demonstrates that dietary supplementation with CCHM during the suckling period improves growth performance, enhances nutrient digestibility, increases rumen fermentation capacity, modulates microbial abundance, and promotes lamb development in *Hu* sheep.

## 1 Introduction

Healthy development in juvenile ruminants lays the foundation for productive performance in adulthood. In livestock production, suckling young ruminants face challenges such as immature gastrointestinal development, growth retardation due to malnutrition, reduced immune function, and intense weaning stress responses (Yang et al., 2015; Mao et al., 2023). Juvenile ruminants, noted for rapid growth and greater adaptability, can achieve improved gastrointestinal development, enhanced somatic growth, reduced weaning stress, and better performance through optimized nutritional interventions during this critical period (Baldwin et al., 2004; Yáñez-Ruiz et al., 2010). Under antibiotic reduction and restriction policies, Chinese herbal medicines (CHM) offer advantages such as low toxicity, minimal antimicrobial resistance, natural origin, multifunctionality, safety, affordability, and environmental sustainability (Bai et al., 2020; Zhang et al., 2024).

CHM contains multiple bioactive substances, including antimicrobial agents, alkaloids, polysaccharides, glycosides, essential oils, tannins, and organic acids (Al-Snafi and Hasham, 2023). They also provide amino acids, minerals, vitamins, pigments, and growth-regulating compounds (Zhang et al., 2025). The rational use of CHM in livestock production has shown multifunctional benefits. Maintaining rumen microecological balance is critical for ruminant productivity and overall health (Su et al., 2024). Studies show that CHM promotes lamb growth, improves immune response, and enhances ruminal function (Tian et al., 2023; Wang et al., 2023). (Zhu et al. 2018) showed that dietary supplementation with a compound containing *Atractylodes macrocephala* and *Astragalus membranaceus* in beef cattle improved nutrient digestibility, increased ruminal enzymatic activity, and modulated microbial structure. A study reported that *Sophora alopecuroides* supplementation in high-concentrate diets regulated fermentation, optimized microbial ecosystems, and improved health in sheep (An et al., 2023). Additionally, phytochemicals and secondary plant metabolites modulate rumen microbiota and activate metabolic pathways to improve fermentation (Peng et al., 2024). However, their exact mechanisms still require further study.

Based on the known effects of CHM in reducing antibiotics use, improving immunity, digestion, and nutrition, and increasing stress tolerance (Zou et al., 2024), this study used a compound formulation of ten botanicals. Based on the recommendation of a Traditional Chinese Medicine practitioner, we have procured the required CHM for this trial from the domestic Chinese market. These include *Astragalus membranaceus, Saposhnikovia divaricata, Atractylodes macrocephala*, and *Raw malt*. The preparation was designed to invigorate the spleen, tonify qi, clear heat, and support gastrointestinal function, thereby enhancing lamb growth and development. To test this hypothesis, the study evaluated CHM-supplemented diets through growth metrics, nutrient digestibility, rumen fermentation, and microbial architecture. The aim was to establish a basis for applying phytogenic compounds in early lamb-rearing strategies.

## 2 Materials and methods

### 2.1 Study site and animal ethics

This study was conducted at Anxin Animal Husbandry in Bachu County, Kashgar City, Xinjiang Uygur Autonomous Region, China (77.82372° E, 39.36478° N). The animal use protocol and experimental procedures involved in this research were approved by the Animal Care and Use Committee of the Feed Research Institute, Xinjiang Academy of Animal Sciences (Approval No.: 20230510).

### 2.2 Experimental material

The compound herbal preparation (CCHM) was composed of ten pharmacopeial ingredients that were commercially obtained, including *Astragalus membranaceus, Saposhnikovia divaricata, Atractylodes macrocephala* (stir-fried), *Hordeum vulgare germinatum, Citrus reticulata pericarpium, Cyrtomium fortunei, Massa Medicata Fermentata, Citri reticulatae Pericarpium Viride, Berberidis Radix*, and *Ostreae Concha Calcinata*. All the above-mentioned traditional Chinese medicines were purchased from the market in Urumqi, China. The aforementioned botanical herbal materials were cut into segments of 5–10 cm in length, air-dried under sunlight, pulverized using a TCM herbal grinder, and sieved through a 60-mesh (250 μm) sieve.These materials were mixed according to specified mass ratios (2:2:1:3:2:2:2:2:3:1, w/w). Untargeted metabolomic profiling was conducted using an ExionLC™ UHPLC system (Sciex) with a Waters ACQUITY UPLC HSS T3 column (1.8 μm, 2.1 × 100 mm). An appropriate amount of sample was added to a pre-chilled methanol: acetonitrile: water (2:2:1, v/v/v) mixture and thoroughly vortex-mixed. Then, it was subjected to power ultrasound treatment at 4 °C for 30 min, followed by stand at −20 °C for 10 min, and finally centrifuged at 14,000 × g for 20 min. The collected supernatant was dried under vacuum. Chromatographic separation was performed using an Agilent 1290 Infinity LC C-18 ultra-performance liquid chromatography (UPLC) column. The column temperature was set to 40 °C, with a flow rate of 0.4 mL/min and an injection volume of 2 μL. Mobile phase A consisted of water, 25 mM ammonium acetate, and 0.5% formic acid, while mobile phase B was methanol. During the experiment, the samples were placed in four separate autosamplers, with quality control (QC) samples arranged separately. Each sample was analyzed using electrospray ionization (ESI) detection in both positive and negative ion modes. Mass spectrometric detection was carried out using a SCIEX TripleTOF^®^ 6600 system. Data acquisition and quantitative analysis were performed with Analyst^®^ TF Software (v1.6.3).

### 2.3 Experimental design and diet composition

Sixty 8-day-old *Hu* lambs (4.64 ± 0.34 kg BW) in good health and with uniform body weight were selected. The lambs of the ewe's second litter are a male lamb and a female lamb. The lambs were randomly assigned to two groups, with three replicates per group and ten lambs in each replicate. Considering gastrointestinal sensitivity and tolerance in suckling lambs. The lambs were randomly assigned to two groups, with three replicates per group and ten lambs in each replicate. Considering gastrointestinal sensitivity and tolerance in suckling lambs, we incorporated the CCHM into the formula and manufactured it into pelletized lamb starter feed, the basal diet was supplemented with 0% (control group, CON) and 0.2% (treatment group, Treat) of the compound herbal additive (w/w)., the basal diet was supplemented with 0% (control group, CON) and 0.2% (treatment group, Treat) of the compound herbal additive (w/w). The experiment lasted for 52 days, including a 7-day acclimation phase and a 45-day formal trial period. Starter feed for suckling lambs was formulated based on the Nutrient Requirements of Meat Sheep (NY/T 816-2021) standard. The ingredient composition and nutritional specifications are shown in Table 1.

**Table 1:** Composition of starter feed and nutrient level of lambs (Dry matter basis).

**Ingredients**	**Content %**	**Nutrientlevels^2^**	**Content %**
Corn	41.42	CP	16.20
Soybean meal	19.00	EE	4.16
Sativa	15.00	Ash	11.29
Premix^1^	5.00	NDF	22.26
Yeast powder	4.00	ADF	8.93
Whey powder	3.80	Ca	1.07
Cottonseed protein	3.00	P	0.51
Glucose	2.90	GE/(MJ/kg)	15.64
Soybean oil	2.00		
CaHPO_4_	1.00		
NaHCO_4_	1.00		
NaCl	1.00		
Lysine	0.45		
Methionine	0.20		
Mold remover	0.10		
Multivitamin	0.10		
Cysteine	0.03		
Total	100.00		

^1^Premixes were provided per kilogram:VA 20000 IU,VD_3_ 10,000 IU,VE 260 mg,C_6_H_5_NO_2_ 250 mg,Cu 180 mg,Fe 2,000 mg,Zn 1,200 mg,Mn 1,000 mg,I 10 mg,Se 7 mg,Co 6 mg,Ca 9 mg,P 1.3 mg,Nacl 5 mg. ^2^The nutritional levels represent measured values.

### 2.4 Feeding and management

Before trial initiation, the experimental pens underwent modification and were thoroughly disinfected. Lambs are vaccinated when ear tagging is performed after birth. To support the postpartum recovery of ewes, a separate housing system was established for lactating dams and their offspring. The lamb pen measures 1.5 m × 3 m, equipped with a feed trough of 50 cm × 20 cm and an automatic water dispenser. The mother-lamb dividing barrier features a 40 cm × 30 cm gated passage for lambs to pass through. Controlled maternal contact protocols were applied as follows: lambs aged 15–22 days were co-housed three times daily for 1 h each (09:00, 13:00, 17:00). Lambs aged 23–44 days had two co-housing sessions (09:00, 17:00). Lambs aged 45–60 days were co-housed once daily at 09:00. Lambs received starter feed at 09:00 and 18:00 daily and had ad libitum access to feed and water between feeding times.

### 2.5 Determination of growth performance

Lambs were weighed for body weight (BW) before morning feeding at 15, 35, 45, and 60 days of age (CON: *n* = 30; Treat: *n* = 30). These data were used to calculate average daily gain (ADG). Daily feed provision and residual amounts were recorded accurately to determine average daily feed intake (ADFI).


$$\begin{array}{l} \text{ADG }\left(\text{g}/\text{d}\right)\\ =\left(\text{Final BW }-\text{ Initial BW}\right)/\text{Experimental duration}\left(\text{d}\right)\\ \text{ADFI }\left(\text{kg}/\text{d}\right)\\ =\left(\text{Total feed offered }-\text{ Feed residues}\right)/\text{Number of lambs} \end{array}$$

### 2.6 Determination of apparent digestibility of nutrients

On day 46, six lambs per group were randomly selected and housed individually for digestibility trials (CON: *n* = 6; Treat: *n* = 6). These trials included a 3-day adaptation period and a 4-day collection period. The acid-insoluble ash (AIA) was used as the internal standard to determine the apparent total digestibility of nutrients, and the measurement was conducted in a muffle furnace at 550°C for 8 h (Furuichi and Takahashi, 1981). During the collection period, fecal and feed samples were collected twice daily (morning and evening) for four consecutive days and immediately stored at −20°C. After the trial, individual feed samples were homogenized by quartering and coning. Fecal sub-samples were preserved in 250 mL amber bottles with 4 M hydrochloric acid for nitrogen fixation. The remaining feces were oven-dried at 65°C to constant weight. The chemical composition was determined using the methods of AOAC in dry matter (DM) number 930.15, ash number 942.05, crude protein (CP) number 992.15 and ether extract (EE) number 920.39 (AOAC International, 2016). The neutral detergent fiber (NDF) and acid detergent fiber (ADF) contents in the samples were determined using the method described by (van Soest et al. 1991).

### 2.7 Determination of rumen fermentation parameters

The lambs were slaughtered at 61 days of age (CON: *n* = 6; Treat: *n* = 6). Before slaughter, lambs were fasted for 12 h and then euthanized humanely through jugular exsanguination. Rumen contents were collected aseptically within 10 min postmortem. A 50 mL rumen fluid sample was filtered through four-layer sterile medical gauze (200 μm pore size). Fresh subsamples were analyzed immediately for pH using a calibrated digital pH meter (Mettler Toledo FE28). The remaining aliquots were snap-frozen in liquid nitrogen and stored at −80 °C. Ammonia nitrogen (NH3-N) concentration was measured spectrophotometrically (UV-1800, Shimadzu) using the Berthelot reaction with alkaline sodium hypochlorite-phenol reagent at 630 nm. Volatile fatty acid (VFA) profiles were quantified by gas chromatography (GC, Agilent 6890N) with flame ionization detection and 2-ethylbutyric acid as internal standard. The injection parameters included a 1 μL split volume with a 20:1 split ratio. VFA separation was conducted on an HP-INNOWax capillary column (30 m × 0.32 mm × 0.5 μm; Agilent HP19091N-213) using helium as carrier gas at 2.0 mL/min constant flow. The FID detector was maintained at 210 °C, with hydrogen and air flow rates set at 40 and 400 mL/min, respectively.

### 2.8 Determination of rumen flora structure

Rumen fluid samples were aliquoted into 5 mL cryovials using the protocol in Section 2.6 and stored at −80 °C for later analysis. Total genomic DNA was extracted from six randomly selected samples per group using the Omega Soil DNA Kit (Cat. No. M5635-02; Omega Bio-Tek, Norcross, GA, USA) according to manufacturer instructions (CON: *n* = 6; Treat: *n* = 6). DNA integrity was checked by 0.8% agarose gel electrophoresis, and concentration was quantified with a NanoDrop™ OneC microvolume spectrophotometer (Thermo Fisher Scientific). The V3–V4 hypervariable regions of bacterial 16S rRNA genes were amplified with universal primers 338F (5′-ACTCCTACGGGAGGCAGCAG-3′) and 806R (5′-GGACTACHVGGGTWTCTAAT-3′). Library construction was conducted with TruSeq^®^ DNA PCR-Free kits. Paired-end sequencing (2 × 250 bp) was performed on the Illumina HiSeq 2500 platform with >80,000 reads per sample. Raw reads were demultiplexed by index sequences, then filtered for quality, denoised, merged, and checked for chimeras using QIIME2 (v2021.11) and the DADA2 plugin. High-quality sequences were clustered into amplicon sequence variants (ASVs) at 100% identity threshold by *de novo* clustering. The raw data were uploaded to the NCBI SRA database (BioProject ID: PRJNA1267695).

### 2.9 Data analysis

Statistical analyses were conducted using SAS 9.4 (SAS Institute Inc.). One-way ANOVA and Duncan multiple range test were applied for group comparisons. Data are presented as mean ± standard error of the mean (SEM). Statistical significance was defined as *P* < 0.05. Microsoft Excel 2021 (Microsoft Corp.) was used for initial data processing.

## 3 Results

### 3.1 Bioactive composition of CCHM

As shown in Table 2, untargeted metabolomic profiling identified ten major bioactive compounds in the herbal formulation. These included Quercetin-3-O-neohesperidoside, Tangeretin, Hesperidin, D-2-Aminobutyric acid, Adenosine, p-Methylbenzaldehyde, Ethyl p-aminobenzoate, Naringenin chalcone, Isoliquiritigenin, and Rhoifolin. These compounds were primarily categorized as flavonoids, flavonoid derivatives, carboxylic acids and their derivatives, nucleotides and their derivatives, and aromatic compounds.

**Table 2:** Top ten active substances in non-target metabolites of compound Chinese herbs.

**Serial number**	**Substance name**	**Classification of substances**	**Content %**
1	Quercetin 3-O-neohesperidoside	Flavonoids	14.21
2	Tangeretin	Flavonoids	9.37
3	Hesperidin	Flavonoid derivatives	8.67
4	D-alpha-Aminobutyric acid	Carboxylic acids and their derivatives	4.84
5	Adenosine	Nucleotide and its derivates	3.39
6	p-Methylbenzaldehyde	Aromatic compounds	2.98
7	Ethyl p-aminobenzoate	Aromatic compounds	2.75
8	Naringenin chalcone	Flavonoids	1.95
9	Isoliquiritigenin	Flavonoid derivatives	1.55
10	Rhoifolin	Flavonoid derivatives	1.33

### 3.2 Effects of CCHM supplementation on growth performance in lambs

As presented in Table 3, dietary supplementation with CCHM significantly influenced growth performance parameters in lambs. Specifically, 0.2% CCHM supplementation resulted in higher body weight at 60 days of age (*P* < 0.05). The treatment group showed significantly increased average starter feed intake from day 15 to 60 compared with the control group (*P* < 0.01). Lambs receiving 0.2% CCHM exhibited greater average daily gain during the 15–60 day period relative to controls (*P* < 0.05).

**Table 3:** Effects of CCHM on growth performance in suckling lambs.

**Items**	**CON group**	**Treat group**	**SEM**	***P*-Value**
Initial BW, kg	5.81	5.93	0.198	0.580
Final BW, kg	11.90	13.38	0.468	0.007
ADFI,g/day	146.87	176.67	6.895	< 0.001
ADG,g/day	132.34	161.96	11.102	0.018

BW, body weight; ADFI, average daily feed intake; ADG, average daily gain.

### 3.3 Effects of CCHM supplementation on apparent digestibility in suckling lambs

As shown in Table 4, the Treat group had significantly higher apparent digestibility of DM, and ADF compared to the Control group (*P* < 0.05). No significant differences were observed in the apparent digestibility of CP and EE between the experimental groups (*P* > 0.05).

**Table 4:** Effects of CCHM on apparent digestibility in suckling lambs.

**Items**	**CON group**	**Treat group**	**SEM**	***P*-Value**
DM	61.01	66.26	1.877	0.049
CP	53.25	56.62	1.416	0.076
EE	74.65	76.67	1.631	0.283
NDF	41.43	46.33	1.807	0.054
ADF	28.61	33.49	1.745	0.049

### 3.4 Effects of CCHM supplementation on ruminal fermentation parameters in suckling lambs

**Figure 1:**
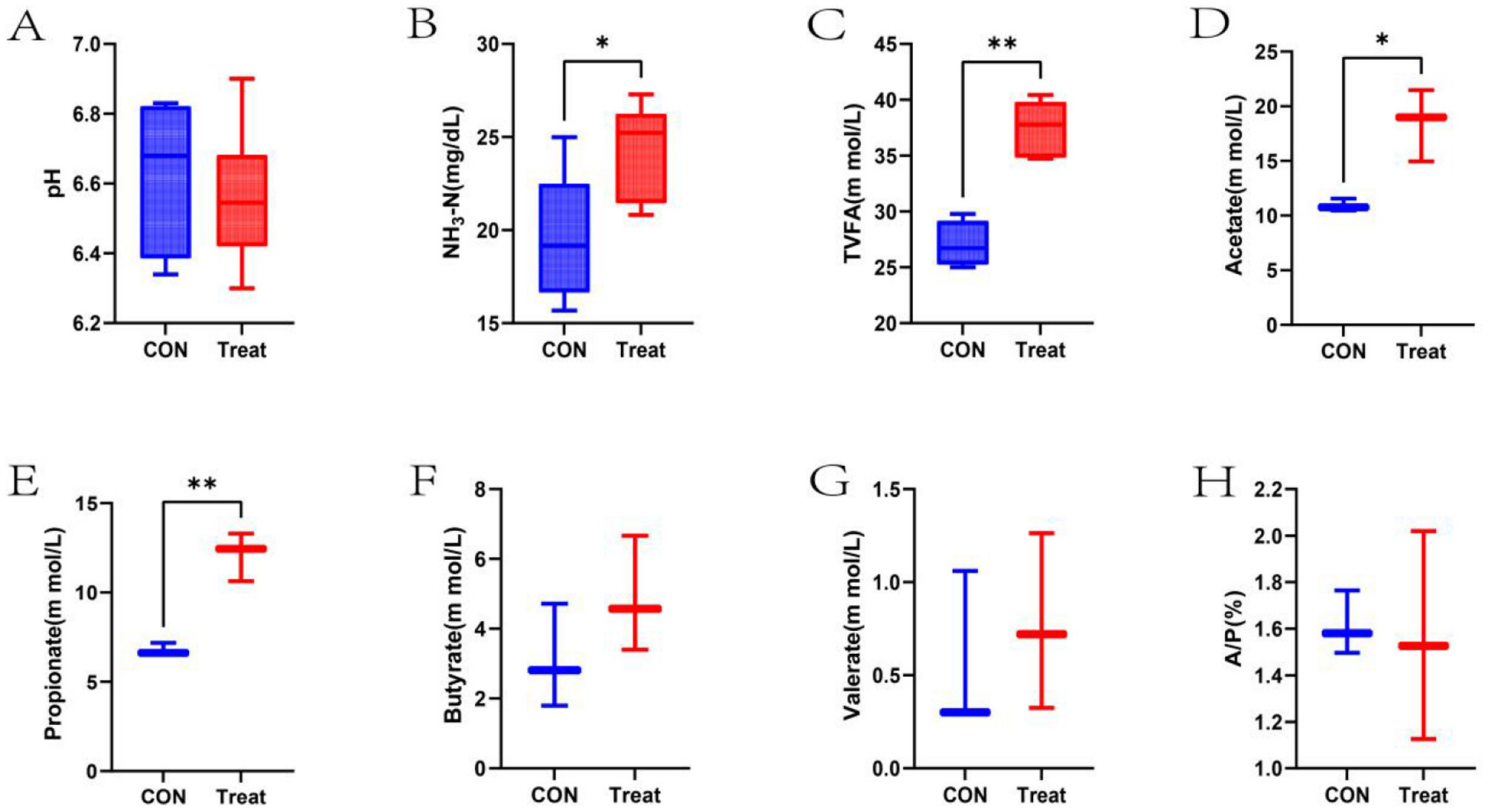
Ruminal fermentation parameters in 60-day-old lambs. **(A)** pH, **(B)** NH_3_-N, **(C)** Total volatile fatty acids, **(D)** Acetate, **(E)** Propionate, **(F)** Butyrate, **(G)** Valerate, and **(H)** Acetate-to-Propionate ratio (A/P) in the CON and Treat groups. *p < 0.05, **p < 0.01.

Ruminal fermentation parameters of lambs in the CON and Treat groups are illustrated in Figure 1. No significant difference was observed in pH values between the CON and Treat groups (*P* > 0.05). The Treat group had significantly higher NH3-N concentration compared to the CON group (*P* < 0.05). TVFA, acetate, and propionate concentrations in the Treat group were significantly elevated relative to the CON group (*P* < 0.05). Butyrate, valerate concentrations, and the acetate-to-propionate ratio showed no statistically significant differences compared to the CON group (*P* > 0.05).

### 3.5 Effects of CCHM supplementation on rumen microbiota and carbohydrate-active enzymes in suckling lambs

**Figure 2:**
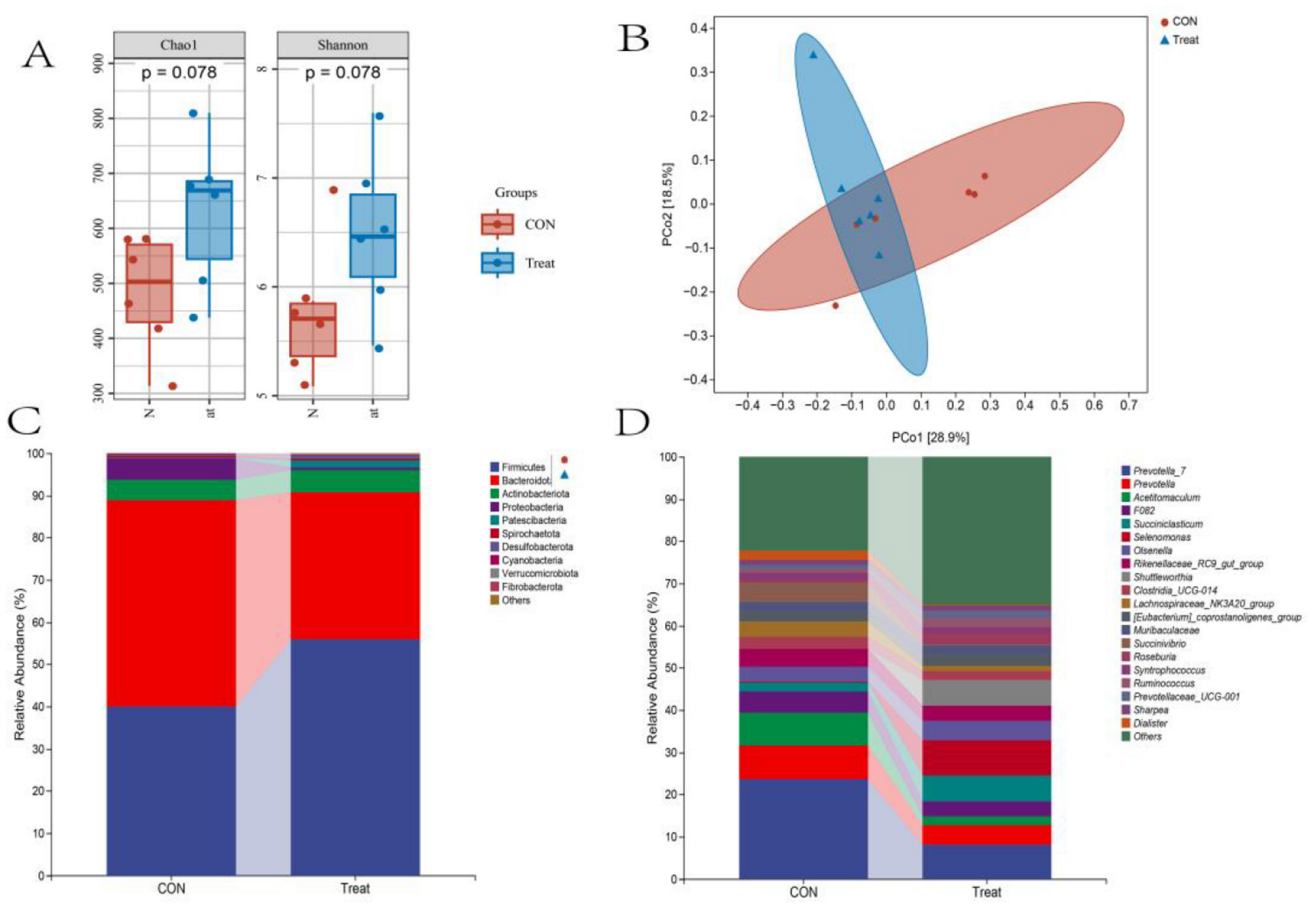
Rumen bacterial diversity and composition in 60-day-old lambs. **(A)** Alpha diversity and **(B)** beta diversity (PCoA) in CON and Treat groups. Relative abundance of the **(C)** top 10 bacterial phyla and **(D)** top 20 bacterial genera.

Alpha diversity analysis revealed no significant difference in community richness (Chao1 index) or diversity (Shannon index) between the CON and Treat groups (*P* = 0.078, Figure 2A). Principal coordinates analysis (PCoA) showed incomplete group separation, with principal component 1 (PC1) explaining 42.3% and PC2 accounting for 14.02% of the total variance (Figure 2B). At the phylum level, the Treat group showed predominant abundances of Firmicutes, Bacteroidota, Actinobacteriota, and Patescibacteria. Among these, Firmicutes, Actinobacteria, and Patescibacteria had higher relative abundance compared to the CON group (Figure 2C). The genus-level analysis identified *Selenomonas, Prevotella_7, Succiniclasticum, Shuttleworthia, Olsenella*, and *Prevotella* as dominant taxa in the Treat group. *Succiniclasticum, Selenomonas, Olsenella*, and *Shuttleworthia* demonstrated significantly greater abundance relative to the CON group (Figure 2D).

**Figure 3:**
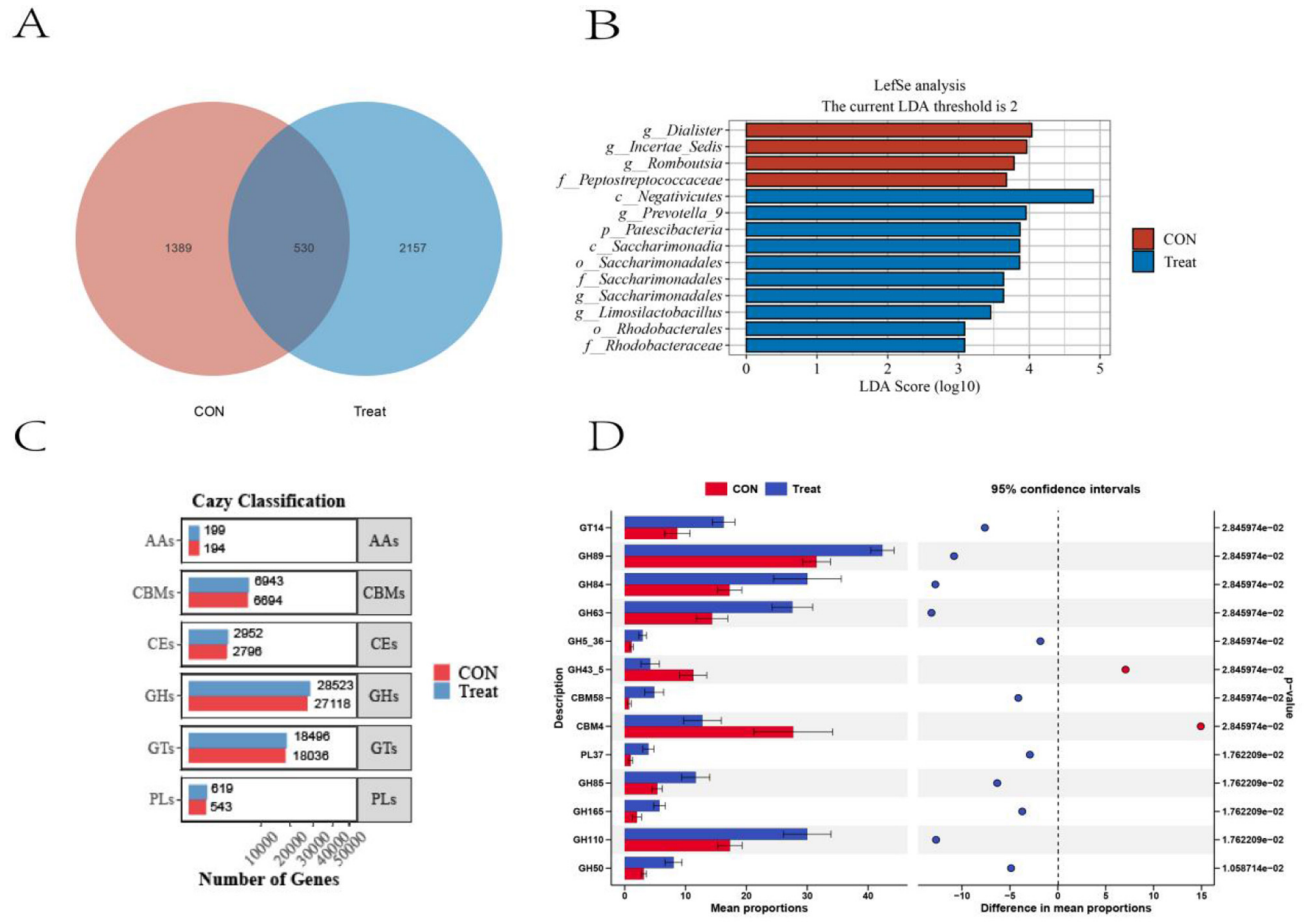
Rumen bacterial ASVs and CAZyme profiles in CON and Treat groups. **(A)** Venn diagram showing the number of shared and group-specific ASVs. **(B)** LEfSe analysis identifying differentially abundant bacterial taxa (*P* < 0.05; LDA score > 2). **(C)** Category distribution of CAZyme profiles in rumen metagenomes. **(D)** Differentially expressed CAZyme families between groups.

A total of 4,076 ASVs were identified in the rumen bacterial communities, with 530 ASVs shared by both groups. The Treat group showed greater ASV richness, with 2,157 group-specific ASVs representing 80.28% of its total ASV composition (Figure 3A). LEfSe analysis (LDA ≥ 2, *P* < 0.05) identified 14 differentially abundant microbial taxa between the two groups. The Treat group was significantly enriched with 10 taxa: Patescibacteria (phylum); Negativicutes and Saccharimonadia (class); Saccharimonadales and Rhodobacterales (order); Saccharimonadaceae and Rhodobacteraceae (family); and *Prevotella_9, Saccharimonadales*, and *Limosilactobacillus* (genus). The CON group was enriched with 4 taxa: Peptostreptococcaceae (family) and Dialister, Incertae Sedis, and Romboutsia (genus) (Figure 3B). Comparative analysis of CAZyme gene abundance in rumen metagenomes between the CON and Treat groups (Figure 3C) revealed six functional categories: glycoside hydrolases (GHs), glycosyl transferases (GTs), carbohydrate-binding modules (CBMs), carbohydrate esterases (CEs), polysaccharide lyases (PLs), and auxiliary activities (AAs). A differential abundance of CAZyme families between the CON and Treat groups was observed (Figure 3D). Relative abundance distribution was as follows: AAs, 0.35% vs. 0.34%; CBMs, 12.09% vs. 12.03%; CEs, 5.05% vs. 5.11%; GHs, 49.97% vs. 49.41%; GTs, 32.58% vs. 32.04%; and PLs, 0.98% vs. 1.07%, for the CON and Treat groups, respectively. Thirteen differentially expressed CAZyme families were identified, including 2 CBMs, 9 GHs, and 1 GT. Two families, GH34-5 and CBM4, were significantly enriched in the CON group. Eleven families, including GT14, GH89, GH84, GH63, GH5-36, CBM58, PL37, GH85, GH165, GH110, and GH50, were enriched in the Treat group.

### 3.6 Relevant heat map analysis

**Figure 4:**
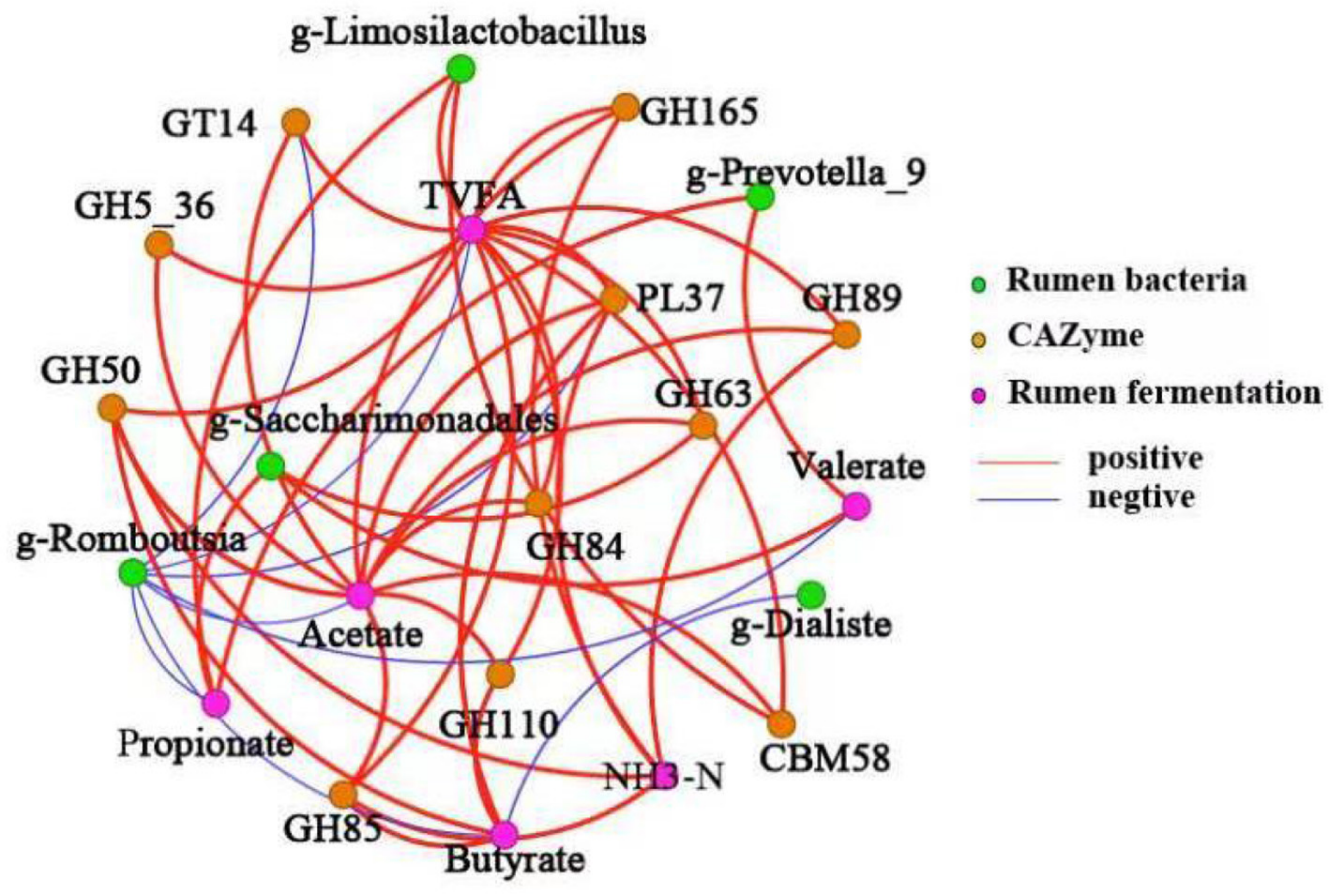
Spearman correlation network among differential rumen microbiota, CAZymes, and fermentation parameters. Line thickness represents correlation strength. Orange-red lines indicate positive correlations; blue lines indicate negative correlations. Node colors: amber for bacterial taxa, purple for CAZymes, and green for fermentation parameters. Significance levels: *P < 0.05; **P < 0.01.

Data in Figure 4 illustrates the Spearman correlation network between differential rumen bacteria, CAZymes, and fermentation parameters. *Prevotella_9* was positively correlated with propionate and valerate concentrations. *Limosilactobacillus* showed significant associations with TVFA, propionate, and CBM58 abundance. *Saccharimonadales* displayed positive correlations with acetate, propionate, valerate, GH63, and GT14. NH3-N levels were positively correlated with butyrate, GH50, GH84, and GH89. TVFA showed significant correlations with acetate, GT14, GH5_36, GH50, GH85, GH84, GH110, GH68, PL37, GH89, and GH165. Acetate concentration was positively associated with GH5_36, GH50, GH85, GH110, CBM58, GH84, GH63, GH89, and PL37. Butyrate levels had positive correlations with GH85, GH50, and PL37.

## 4 Discussion

### 4.1 Growth performance response to CCHM supplementation in suckling *Hu* lambs

Animal growth and development are affected by breed characteristics, genetic factors, nutritional input, and diet palatability. ADG acts as a key indicator of livestock growth dynamics, while ADFI reflects feed preference and acceptability (Bokelman et al., 2015). Previous studies have reported the growth-promoting properties of Chinese herbal feed additives in livestock production (Zhang et al., 2020). The CCHM used in this study was characterized by untargeted metabolomics. Quercetin, tangeretin, and hesperidin were identified as predominant bioactive constituents. Guo et al. found that dietary quercetin supplementation significantly enhanced both ADFI and ADG in lambs (Guo et al., 2018). Our results were consistent with these findings, showing that 0.2% (w/w) CCHM increased lamb ADFI effectively. (Hao et al. 2020) determined that 5–10 g/kg Astragalus root powder was optimal for enhancing ADG in lambs, with diminished effects at higher concentrations. Lambs fed a 0.2% CCHM-supplemented diet demonstrated 20.29% and 22.38% greater ADFI and ADG, respectively, compared with the CON group. This improvement may be linked to flavonoid-mediated modulation of gut microbiota and enzymatic activation (Kasahara et al., 2025), which collectively enhanced nutrient use efficiency and metabolic partitioning. These changes ultimately promoted ADG.

### 4.2 Impact of CCHM on apparent digestibility in suckling lambs

Apparent nutrient digestibility indicates the effectiveness of nutrient absorption and directly influences growth performance in ruminants. The inclusion of CHM in animal feed enhances digestive efficiency and nutrient utilization, as its bioactive compounds optimize dietary balance and improve feed conversion ratios (Liu et al., 2023). (Lunsin et al. 2021) demonstrated a correlation between DM degradation rate and feed intake in ruminants. In this study, both intake and DM degradation rates were significantly increased in the Treat group, reflecting parallel improvements in nutrient use and growth. (Du et al. 2018) reported that supplementing *Astragalus membranaceus* and *Artemisia argyi* enhanced the apparent digestibility of DM, OM, CP, and NDF in *Mongolian* sheep. (Song et al. 2014) observed that 10 g/kg supplementation with *Atractylodes macrocephala, Astragalus membranaceus*, and *Eriobotrya japonica* significantly improved NDF and ADF digestibility in heat-stressed beef cattle. In this study, CCHM supplementation promoted fibrolytic bacteria and improved fiber degradation efficiency through ruminal cellulolytic modulation (Du et al., 2018). This effect was reflected by increased ADF digestibility in the Treat group. The lack of significant differences in Ether Extract digestibility was consistent with findings by (Wu et al. 2022) and (Zhong et al. 2012), possibly due to the dose-dependent effects of phytogenic compounds.

### 4.3 Influence of CCHM supplementation on ruminal fermentation in suckling lambs

Ruminal pH functions as a key indicator of fermentation homeostasis. Physiological variation is regulated by dietary composition and metabolic status (Fu et al., 2024). All lambs maintained the pH within this range, indicating that CCHM supplementation had no adverse impact on fermentation. NH3-N concentration reflects microbial nitrogen metabolism, indicating a balance between proteolysis and microbial utilization efficiency (Ma et al., 2024). Dietary proteins are degraded to NH3-N, which is assimilated by microbes to synthesize microbial crude protein (MCP) (Lv et al., 2024). Optimal NH3-N levels (5–25 mg/dL) support microbial growth and improve MCP synthesis through efficient nitrogen recycling (Sun et al., 2018). Our data showed that CCHM supplementation elevated NH3-N levels, suggesting improved nitrogen use through microbial metabolic regulation. Ruminal VFAs act as microbial-derived signaling molecules that regulate epithelial development and energy metabolism (Zeineldinms et al., 2018; Shen et al., 2021). Ruminants primarily rely on hepatic gluconeogenesis for glucose needs, with over 80% of adult sheep glucose turnover originating from this pathway (Bergman et al., 1970). Propionate contributes 27–59% of glucose carbon precursors (Amaral et al., 1990). The acetate-to-propionate (A/P) ratio reflects fermentation patterns, as microbial substrate selectivity influences digestive efficiency and systemic metabolism (Vargas et al., 2023). (Wang et al. 2018) reported that CCHM supplementation in *in vitro* systems shifted fermentation toward acetate. Elevated propionate concentration in the Treat group indicated enhanced energy efficiency, consistent with improved ADG. Variability in flavonoid subclasses (e.g., flavones vs. flavonols) affects VFA profiles, revealing structure-activity relationships. (Ehsan et al. 2013) found that 4.5% DM quercetin did not alter TVFA, acetate, or propionate *in vitro*. In contrast, mulberry leaf flavonoids enhanced TVFAs and AA levels in beef cattle (Hassan et al., 2020). Our findings demonstrated that 0.2% (w/w) CCHM supplementation increased ruminal TVFAs, thereby improving energy availability to support growth in *Hu* lambs. Flavonoids have been shown to enrich cellulolytic bacterial populations in finishing bulls fed a silage–mulberry diet. Their derivatives also enhance microbial metabolism in mature ruminants (Li et al., 2017). Increased propionate likely resulted from a greater abundance of propionate-producing bacteria, which reduced the A/P ratio through microbial community restructuring (Berger et al., 2012). Higher propionate production correlates with improved performance, as supported by our results and previous reports.

### 3.4 Modulation of rumen microbiota by CCHM in suckling lambs

The gastrointestinal microbiota in ruminants is closely associated with host metabolism, feed efficiency, and nutrient absorption (Shabat et al., 2016). Lambs supplemented with flavonoid-rich herbal additives showed increased trends in ruminal Chao1 and Shannon indices. This observation aligns with the findings of (Zhan et al. 2017) showing that alfalfa flavonoids enhanced microbial diversity in dairy cows. Bacteroidetes and Firmicutes are dominant phyla in ruminant microbiota. These groups drive nutrient metabolism through polysaccharide degradation and fatty acid processing while maintaining microbial ecosystem balance (Pinnell et al., 2022; Lapébie et al., 2019). Under experimental conditions, the Treat group retained core phyla Bacteroidetes, Firmicutes, and Proteobacteria, suggesting that CCHM supplementation preserved core microbial architecture. Firmicutes play essential roles in energy metabolism by degrading cellulose, producing short-chain fatty acids, and facilitating microbial cross-feeding (Chen et al., 2025). Increased Firmicutes abundance is correlated with improved ADG and feed efficiency indices (Min et al., 2019).

Rumen microbiota hydrolyzes β-glycosidic bonds in quercetin-3-O-rutinoside, releasing bioactive quercetin and enhancing its absorption (Berger et al., 2012). (Miron et al. 2002) found that citrus pulp substitution for corn starch enhanced NDF degradation and feed utilization by optimizing fibrolytic activity. (Paniagua et al. 1972) showed that citrus flavonoid supplementation modulated rumen microbiota by stimulating beneficial bacterial proliferation. The increased Firmicutes abundance and ADG in the CCHM group agree with these studies, potentially due to quercetin and citrus flavonoids enhancing fibrolytic bacteria and upregulating cellulolytic enzymes, which improved nutrient utilization. Ketone bodies and polyphenols may influence microbiota composition by promoting beneficial taxa, suppressing pathogens, and maintaining microbial balance, thereby supporting nutrient assimilation and host development (Mao et al., 2024). Phytochemicals from Chinese herbs can regulate microbiota bidirectionally by stimulating probiotics and inhibiting harmful bacteria (Chen et al., 2021). Elevated Proteobacteria abundance is regarded as a dysbiosis marker (Shen et al., 2023). Reduced Proteobacteria in CCHM-fed lambs suggests suppression of pathogenic taxa via flavonoids, possibly reducing metabolic disorder risk. *Succiniclasticum*, a Gram-negative bacterium, ferments carbohydrates and starch, converting succinate to propionate through the acrylate pathway. Enrichment of propionate-producing bacteria and related genes is linked with improved feed efficiency in ruminants (Shabat et al., 2016). The increased abundance of *Succiniclasticum* in the Treat group indicates improved microbial energy metabolism, contributing to better lamb growth.

As key facilitators of plant fiber degradation, rumen microbiota contain cellulolytic microorganisms and CAZymes that support anaerobic fermentation, converting cellulose, hemicellulose, and lignin into monosaccharides for VFA production (Liang et al., 2021). The major CAZyme families include GHs, GTs, CBMs, CEs, PLs, and AAs. GH families are widely used in biotechnology and biomedicine (Neves et al., 2021). The abundance of GHs in the Hu sheep rumen microbiome, as reported by (He et al. 2019). demonstrated strong fibrolytic capacity, particularly through GH3, GH5, and GH9 families, which are critical for carbohydrate degradation. In this study, the Treat group showed significantly higher GH5-36 gene abundance than CK. This result may reflect stimulation of microbial proliferation by flavonoid and alkaloid components in CCHM, demonstrated by increased Firmicutes and Ruminococcus abundance and enhanced secretion of cellulase and hemicellulase.

GH89 is known as a lysosomal enzyme for heparan sulfate degradation in the human digestive system (Shimada et al., 2015), but its function in ruminant microbiomes remains underexplored. (Fatemeh et al. 2021) identified GH89 in the Verrucomicrobia phylum, where all *Akkermansia* members expressed the enzyme. As a dominant genus in Verrucomicrobia, *Akkermansia* enhances intestinal barrier function and prevents gut permeability disorders. Concurrent increases in ruminal GH89 gene abundance and Verrucomicrobia population were observed, suggesting intestinal health benefits from CCHM, despite unchanged *Akkermansia* levels. GH110 belongs to a specialized group of α-galactosidases with strict substrate specificity for α-1,3-linked galactose residues in polysaccharides and oligosaccharides [58] (McGuire et al., 2020). As annotated in the CAZy database (https://www.cazy.org/), GH84 encodes N-acetylglucosaminidases that hydrolyze β-1,4-glycosidic bonds in chitin-containing plant cell walls, providing microbial access to carbon and nitrogen. Elevated CAZyme gene abundance in CCHM-fed Hu lambs suggests a transition from milk reliance to roughage digestion. This shift was facilitated by enhanced energy metabolism and rumen microbiota maturation.

Dietary supplementation with CHM induced multiple changes in rumen microbiota, CAZyme profiles, and fermentation parameters. *Prevotella_9*, a keystone taxon in the rumen ecosystem, performs various roles in carbohydrate metabolism, proteolysis, and short-chain fatty acid biosynthesis. *Limosilactobacillus* converts soluble sugars to lactate, which acts as a propionate precursor through cross-feeding with secondary fermenters. The observed positive correlations between *Prevotella_9, Limosilactobacillus*, and propionate may result from their cooperative cellulose degradation, generating metabolites that drive propionogenesis. CAZyme families including GH50, GH84, and GH165 were significantly correlated with VFA production. Specifically, GH50-mediated hydrolysis of cellulose and hemicellulose increased monosaccharide availability, supporting microbial VFA synthesis (Ding et al., 2014). Functional relationships among *Saccharimonadales*, CAZymes, and VFA profiles require further study, as *Saccharimonadales* are poorly characterized in ruminant microbiomes. The integrated mechanism suggests that *Prevotella_9* and *Limosilactobacillus* enhance VFA bioproduction by mobilizing enzymatic substrates, thereby supporting lamb growth performance.

## 5 Conclusion

This study showed that supplementation with CCHM improved growth performance and nutrient digestibility in *Hu* lambs. These outcomes were linked to enhanced ruminal fermentation, driven by microbial restructuring and elevated enzymatic activity. Increased cellulose hydrolysis boosted propionate production, supporting better energy utilization and growth. Microbial analysis revealed the enrichment of fibrolytic taxa such as Firmicutes, Actinobacteria, Patescibacteria*, Succiniclasticum, Selenomonas*, and *Olsenella*. Overall, CCHM strengthened rumen fermentation, improved nutrient digestion, balanced microbial composition, and promoted lamb development.

## Data Availability

The datasets presented in this study are publicly available. This data can be found here: https://www.ncbi.nlm.nih.gov/, accession PRJNA1267695.
